# Bush Blitz aids description of three new species and a new genus of Australian beeflies (Diptera, Bombyliidae, Exoprosopini)

**DOI:** 10.3897/zookeys.150.1881

**Published:** 2011-11-28

**Authors:** Christine L. Lambkin, Justin S. Bartlett

**Affiliations:** 1 Entomology, Queensland Museum, PO Box 3300, South Brisbane, Queensland, Australia 4101; 2 DEEDI Entomology, Ecosciences Precinct, GPO Box 46, Brisbane, Queensland, 4001

**Keywords:** *Ngalki*, *Palirika*, *Larrpana*, *Munjua*, *Balaana* genus-group, phylogenetic analysis, cybertaxonomy, Scratchpads, Morphbank

## Abstract

Bush Blitz is a three-year multimillion dollar program to document the plants and animals in hundreds of properties across Australia’s National Reserve System. The core focus is on nature discovery – identifying and describing new species of plants and animals. The Bush Blitz program has enabled the collection and description of beeflies (Diptera, Bombyliidae) from surveys in Western Australia and Queensland. Three new species of Australian beeflies belonging to the Exoprosopini are described; *Palirika mackenziei* Lambkin **sp. n.**, *Palirika culgoafloodplainensis* Lambkin **sp. n.**, and *Larrpana bushblitz* Lambkin **sp. n.** Phylogenetic analysis of 40 Australian exoprosopine species belonging to the *Balaana* generic-group Lambkin & Yeates 2003 supports the placement of the three new species into existing genera, and the erection and description of the new genus *Ngalki* Lambkin **gen. n.** for *Ngalki trigonium* (Lambkin & Yeates 2003) **comb. n.** Revised keys are provided for the genera of the Australian *Balaana* genus-group and the species of *Palirika* Lambkin & Yeates, 2003 and *Larrpana* Lambkin & Yeates 2003. With the description of the three new species and the transferral of *Munjua trigona* Lambkin & Yeates 2003 into the new genus *Ngalki* Lambkin **gen. n.**, three genera are rediagnosed; *Munjua* Lambkin & Yeates 2003, *Palirika* and *Larrpana*.

## Introduction

While there are more than 140,000 published species in Australia, more than 40 per cent of continental Australia has never been comprehensively surveyed by scientists. This research was supported through funding from the Bush Blitz species discovery program, a partnership between the Australian Government, BHP Billiton and Earthwatch Australia. This innovative partnership harnesses the expertise of many of Australia’s top scientists from museums, herbaria, universities, and other institutions and organisations across the country. Bush Blitz is expected to uncover hundreds of new species and provide baseline scientific data that will help us protect our biodiversity for generations to come.

This paper describes three species of beeflies from the Exoprosopini (Diptera, Bombyliidae, Anthracinae); two captured during Bush Blitz surveys and a third species collected from south-western Queensland (Qld). All three species belong to genera recently described (*Palirika* Lambkin & Yeates 2003 and *Larrpana* Lambkin & Yeates 2003) in a large revisionary monograph (Lambkin et al. 2003) and therefore can be described reasonably easily as all collected material has been examined recently, and the context for their description is in place.

The beeflies belong to the Family Bombyliidae, a very large, cosmopolitan family of stoutly built flies, mostly with very characteristic venation. Almost 5000 species have been described worldwide (Evenhuis and Greathead 1999) and around 370 have been described from Australia, with many more species awaiting description (Yeates and Lambkin 2006). Nine of the 15 recognised subfamilies (Yeates 1994) are found in Australia, and a key to these subfamilies is available (Lambkin et al. 2003). Most Australian species belong to the subfamilies Bombyliinae, Anthracinae and Lomatiinae. The Anthracinae are well represented in Australia, mainly by the cosmopolitan *Anthrax* Scopoli 1763, *Ligyra* Newman 1841, *Villa* Lioy 1864, and a number of endemic genera including *Palirika* and *Larrpana* (Yeates and Lambkin 1998; Lambkin et al. 2003). Of the seven anthracine tribes, three (Villoestrini, Prorostomatini, Aphoebantini) are not found in Australia. Keys to the four tribes of the Anthracini occurring in Australia are available (Lambkin et al. 2003). The tribe Xeramoebini is represented in Australia by only two, still undescribed, species of *Petrorossia* Bezzi 1908. There are 28 species of Australian Villini in the genera *Villa*, *Exechohypopion* Evenhuis 1991 and *Lepidanthrax* Osten Sacken 1877(Evenhuis and Greathead 1999).The Anthracini is represented by 34 described species in the genera *Anthrax*, *Brachyanax* Evenhuis 1981, and *Thraxan* Yeates & Lambkin 1998(Yeates and Lambkin 1998). Based on the phylogenetic analyses the Australian Exoprosopini was expanded to ten genera containing 65 species, including seven new genera for 42 species in the *Balaana* genus-group Yeates & Lambkin (Lambkin et al. 2003).

Australian exoprosopines are large beeflies of diverse and striking appearance (Figs 4C, 6D, 7B) with wings usually bearing distinct hyaline and black patterns. Like most bombyliids, adult Australian exoprosopines are well covered in long, dense, coloured hairs arranged in patterns, often in stripes across the dorsal surface of the abdomen, leading to their common name of beeflies. In Australian exoprosopines, like other members of the Anthracinae, many of the long hairs, especially on the dorsal surface, are modified into short, broad, flattened scales, often in contrasting stripes. The scales may be erect or upstanding, producing a “fluffy” appearance as in *Larrpana bushblitz* Lambkin sp. n. (Fig. 7A). Sometimes the dorsal scales are tightly adpressed, producing a smooth, often shiny appearance as in *Palirika* (Fig. 6D). Some beeflies have some scales or hairs that are reflective, appearing shining gold or brilliantly silver as on the terminal tergites of the male anthracine *Anthrax maculatus* Macquart 1846 (Yeates and Lambkin 1998). While many beeflies have vestiture (hairs or scales) that is shiny, only the endemic Australian genus *Palirika* has metallic, reflective scales for which the colour of the reflected light is different from the colour of the scales. In this genus black scales on the dorsal surface of the face, thorax, and abdomen may be iridescent and refractive, and reflect green, blue, maroon or purple colours (Fig. 6C, D). The reflectivity may be very dull, almost dark as in *Palirika mackenziei* Lambkin sp. n. (Fig. 6C, D), or highly reflective and bright (Lambkin et al. 2003).

Adult Australian exoprosopines favour warm, sunny localities, especially in the more arid regions. Most have a strong, hovering flight, and are commonly taken from blossom, or sitting on patches of bare earth. Adults are pollen and nectar feeders, and many are important pollinators of native plants. Many species can be collected congregating on hilltops, demonstrating a landmark-based mating system (Lambkin et al. 2003). Very little is known about the life histories of Australian exoprosopines, but some larvae are hyperparasites, parasitising prepupal instars of Hymenoptera that, in turn, are parasitising Coleoptera (Yeates et al. 1999).

This paper describes three new species of exoprosopine beeflies; two captured during Bush Blitz surveys and a third species collected from south-western Qld.

**Figure 1.**
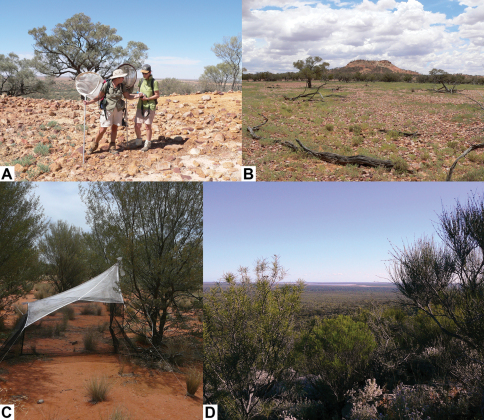
Collection sites. **A** CLL showing Robyn Mackenzie the single female specimen of *Palirika mackenziei* sp. n. collected hill-topping on the summit of Tompilly Hill in late December 2007 **B** Tompilly Hill, a jump up on Plevna Downs, in extremely arid south-western Queensland **C** A single male specimen of *Palirika culgoafloodplainensis* sp. n. was collected during a Bush Blitz survey from this Malaise trap, 7 km NNW Toulby Gate on Culgoa Floodplains National Park (NP) on the Queensland/New South Wales Border, 134 km WSW Dirranbandi **D** Forrest lookout on Karara Pastoral Lease 213 km ESE of Geraldton in Western Australia, where two male specimens of *Larrpana bushblitz* sp. n. were hand netted hill-topping by CLL in September 2009 during a Bush Blitz survey. Photographs A and B by N. Starick, QM.

*Palirika mackenziei* sp. n. wascollected from the large grazing property, Plevna Downs, owned by the Mackenzie family, 63 km west of Eromanga, in extremely arid south-western in late December 2007. While accompanied by Noel Starick (QM volunteer) and Robyn Mackenzie, CLL hand netted a single female specimen (Fig. 6C, D) hill-toppingon the summit of Tompilly Hill (Fig. 1A, B), a jump up on Plevna Downs. This species was unlike any other *Palirika* collected; smaller and darker in both body and wing infuscation.

Four male specimens (Fig. 7A, B) of *Larrpana bushblitz* sp. n. were hand netted by CLL from Karara Pastoral Lease in Western Australia, hill-topping onForrest lookout (Fig. 1D), 24.4km SE Boiada Camp and on a nearby hilltop 23.5km ESE Boiada Camp during the Bush Blitz survey co-organised by WAM on Charles Darwin Reserve, Karara, Lochada and Kadji Kadji Pastoral Leases, 213 km ESE of Geraldton, in September 2009. This species appeared similar to the two male specimens of *Larrpana zwicki* Lambkin & Yeates 2003 collected only near Windorah (Lambkin et al. 2003).

*Palirika culgoafloodplainensis* Lambkin sp. n. was collected from Culgoa Floodplains National Park (NP) on the Queensland/New South Wales Border, 134 km WSW Dirranbandi, during the Bush Blitz survey of Culgoa Floodplains NP Qld, Culgoa NP and Ledknapper Nature Reserve (NR) NSW (NSW) organised by CLL and Noel Starick from QM between November 2009 and June 2010. A single male specimen (Fig. 4C, D) was sorted by QM volunteer John Purdie from a Malaise trap sample from 7 km NNW Toulby Gate (Fig. 1C) on Culgoa Floodplains National Park (NP). Malaise and Pitfall traps had been set at four sites on Culgoa Floodplains by CLL, Noel Starick and NP Ranger Cheryn Kelly in November 2009 as part of the Bush Blitz survey. The rangers had agreed to take monthly samples until we could return. This specimen was from a Malaise trap that had been reset on the 20th January 2010 by Ranger-In-Charge (RIC) Andy (Keith) Coward. Because of significant rain, the rangers were unable to return to take another sample until the 19^th^ March. Subsequent flooding in March and April 2010 prevented access to the survey areas until mid-May when CLL, Noel, Rhys Smith (QM volunteer) and rangers Andy and Megan Simpson retrieved the Culgoa Floodplains NP traps. This species was similar to *Palirika bouchardi* Lambkin & Yeates 2003 that has been extensively collected from arid areas of central and western Australia from all states except South Australia.

**Figure 2.**
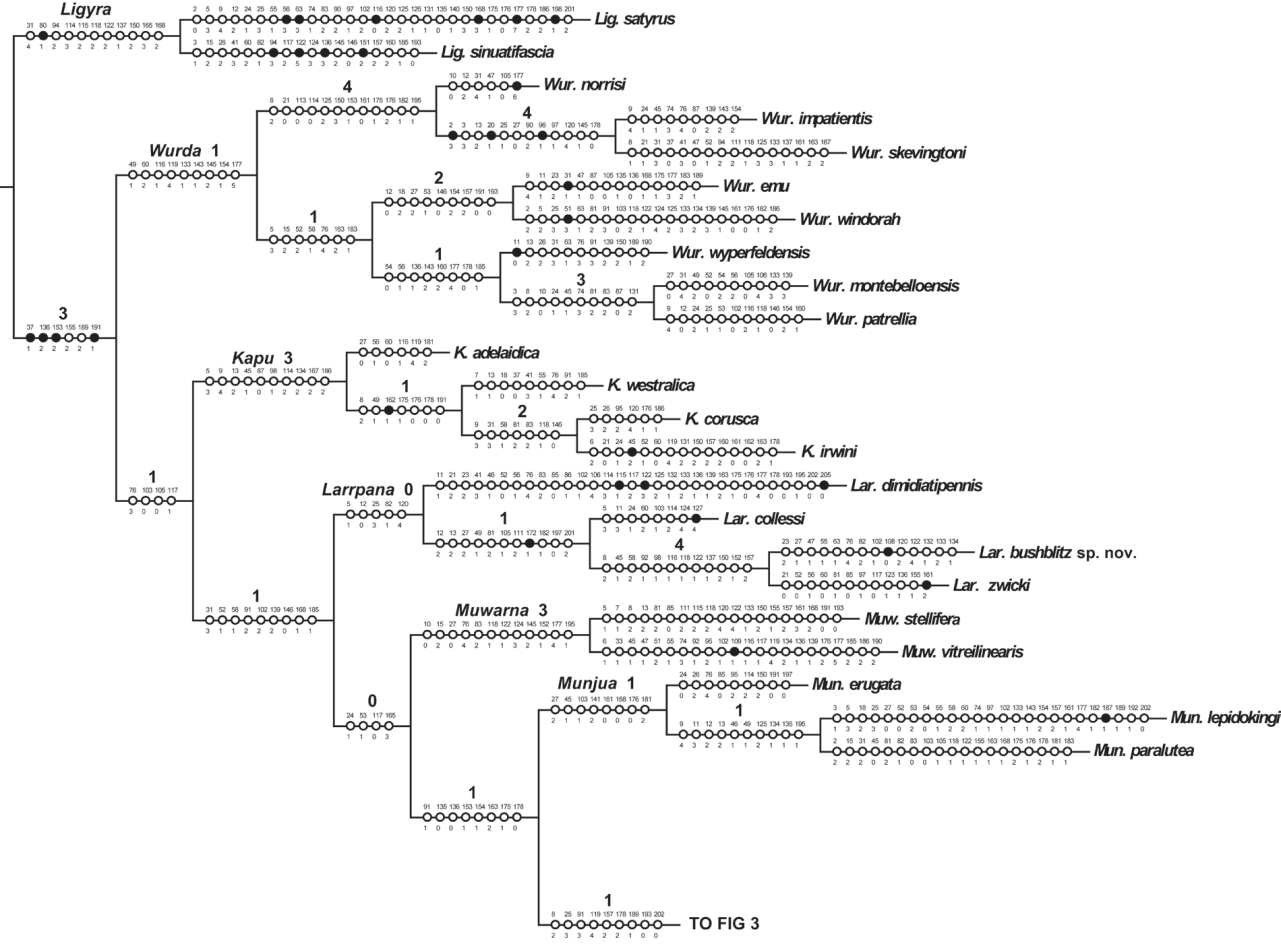
One of five most parsimonious cladograms (931 steps, CI = 0.24, RI =0.47). Part 1. Black circles = unique character changes, open circles = homoplasious changes. Bremer supports over branches.

**Figure 3.**
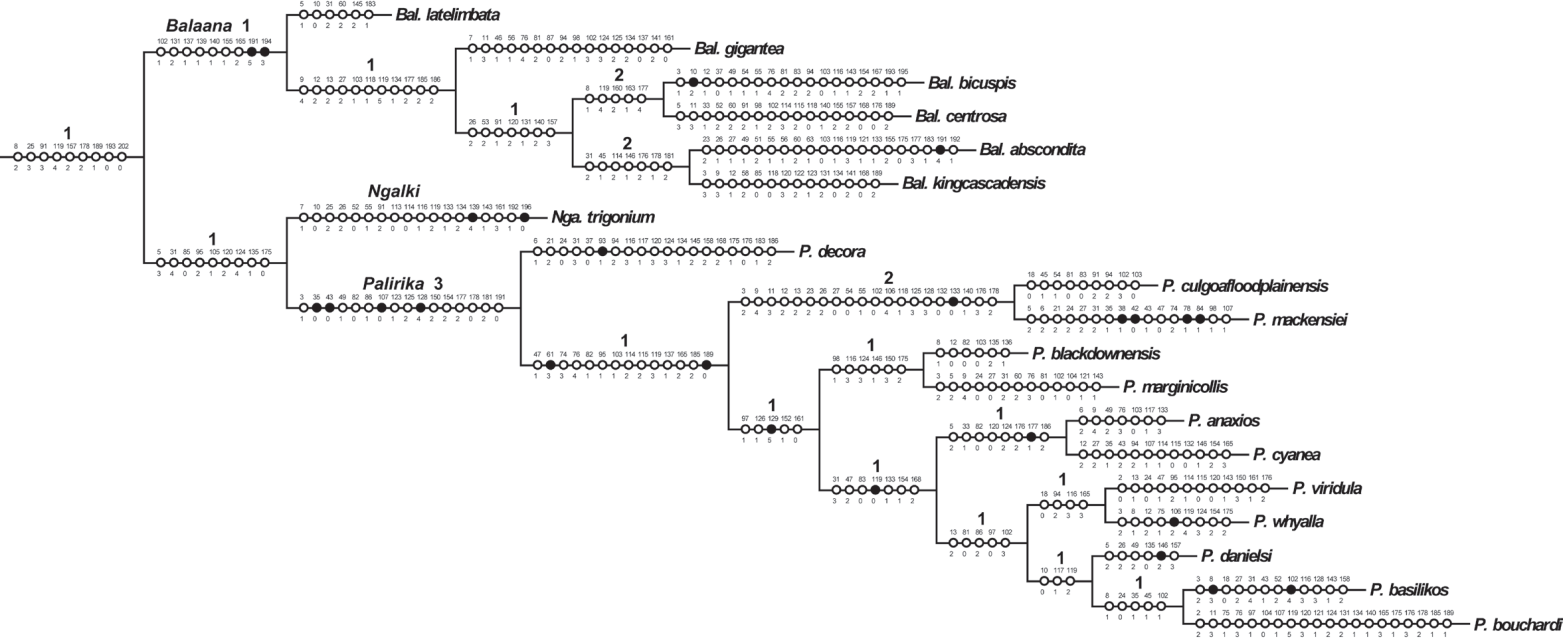
One of five most parsimonious cladograms (931 steps, CI = 0.24, RI =0.47). Part 2. Black circles = unique character changes, open circles = homoplasious changes. Bremer supports over branches.

Previous phylogenetic analysis of the worldwide Exoprosopini showed that the Australian bombyliids that were previously placed in *Exoprosopa* Macquart 1840, belonged to the monophyletic *Balaana* group of genera, sister to the Australian *Ligyra* (Lambkin et al. 2003). Phylogenetic analysis of 207 morphological characters of the *Balaana* group of genera led to the description of seven new genera for 42 species in that genus-group in Lambkin et al. (2003). Phylogenetic analysis of the same 207 morphological characters scored for two *Ligyra* outgroup taxa and 40 Australian species belonging to the *Balaana* generic-group supports the placement of the three new species into existing genera, and the erection of the new genus *Ngalki* Lambkin gen. n. for *Ngalki trigonium* (Lambkin & Yeates 2003) comb. n. (Figs 2, 3).

Revised keys are provided for the genera of the Australian *Balaana* genus-group and the species of *Palirika* and *Larrpana*. The three new species are fully described; with diagnoses, distribution maps, and images of both external characters and dissected genitalia. The new genus *Ngalki* is described with diagnosis, and images of both external characters and dissected genitalia. With the description of three new species and the transferral of *Munjua trigona* Lambkin & Yeates 2003 into the new genus *Ngalki*, three genera are rediagnosed; *Munjua* Lambkin & Yeates, *Palirika* and *Larrpana*.

We attempted to use cybertaxonomic tools to produce this paper as had been used to streamline taxonomic publication of new fly species by Winterton (2009), Brake and von Tschirnhaus (2010), Blagoderov et al. (2010b), and Winterton and Gaimari (2011). Attempts to use the morphological phylogenetic matrix to produce natural language descriptions provided only clumsy, inadequate descriptions. Using Blagoderov et al. (2010a) as a guide we completed automatic generation of the manuscript within a Virtual Research Environment (Scratchpads). As the publication module in Scratchpads is still under development, semantic enhancements, and parallel release of the publication on paper and on-line accompanied with registration of new taxa with ZooBank (http://www.zoobank.org/) as per the recent proposed amendment to the *International Code of Zoological* nomenclature for a universal register for animal names (Polaszek et al. 2005a, 2005b; ICZN 2008) were completed through submission of a Microsoft Office Word 2003 document to ZooKeys.

## Methods

### Taxonomic Methods

The following collection acronyms are used in the text: Australian Museum, Sydney, New South Wales, Australia (AM); Queensland Museum, South Brisbane, Queensland (QM); Western Australian Museum, Perth, Western Australia (WAM). Numbers quoted with individual specimens are unique identifiers (e.g. WAM 82396, T152479 (QM), K 253702 (AM)) from the respective institutions database and are attached under each specimen on a white label. A single hind leg was removed from one specimen of each species and placed into absolute ethanol for frozen tissue storage at QM for future DNA extraction. Those samples were given a tissue number (e.g. A007534) that was entered into the QM Vernon database and attached under each specimen on a yellow label.

For explanation of morphological abbreviations, see Appendix 1.

The genitalia of each species were prepared by dissecting the terminal abdominal segments and then placing in cool 10% KOH overnight. Following maceration the specimen was washed, and then dissected in distilled water. Dissected genitalia were placed in alcohol for microscopic examination and into K–Y^®^ Jelly for photography. All dissected parts from a specimen were placed in a genitalia vial containing glycerine which was pinned beneath the identification label.

Images were taken of the whole fly, external features, and dissected genitalia. A series of multiple-focal-depth digital images were taken using a Canon EOS 500D digital camera fitted, via a Leica 10446175 1x SLR Projection Lens, to a Leica MZ6 stereo dissecting microscope, and combined into a high resolution serial montage image using Helicon Focus v.5.2 Pro (Kozub 2011) or Zerene Stacker v.1.02 (Littlefield 2011). Higher-resolution digital images were deposited in Morphbank (www.morphbank.net). Separate collections of images were created for each species in Morphbank where each collection receives a unique identifier and associated URL. The URL links to the Morphbank collections have been embedded within the descriptions for each species. Images were assembled into plates using Adobe Photoshop C S5 version 13.0.3 (Adobe Systems, 2010b) and Adobe Illustrator C S5 version 15.0.2 (Adobe Systems, 2010a). Those samples were given a photograph number (e.g. PS1714) that was entered into the QM Vernon database and attached under each specimen on a purple label.

Distribution maps were produced using ArcView GIS version 3.1 (ESRI 1998).

We intended to use cybertaxonomic methods to document these newly discovered Australian beeflies, enabling descriptions of the three new species to be generated using web resources to populate electronic documents through links to Morphbank, Life Science Identifiers, and Zoobank as had been done by Winterton (2009) whose revision serves as an example for making taxonomic description and key development more efficient by avoiding redundancy in data handling and using digital media. We hoped to complete taxonomic descriptions using a character matrix in Structured Descriptive Data format developed in Lucid Builder to simultaneously generate natural language descriptions and a key. However we encountered problems transferring the compiled phylogenetic data matrix to Lucid. Instead MacClade 4 (Maddison and Maddison 2003) was used to generate natural language descriptions based on a phylogenetic matrix including 413 phylogenetic (morphological) and phenetic (colour) characters. The resultant descriptions were clumsy and inadequate. Instead, we developed descriptions in Microsoft Office Word 2003 based on the electronic versions of closely related described species from Lambkin et al. (2003).

Several initiatives around the world have been developing tools to bring revisionary taxonomy to the web. Recent examples include software produced through the CATE (Creating a taxonomic e-science, http://www.cate-project.org), EDIT (European Distributed Institute of Taxonomy, http://www.e-taxonomy.eu) and the Australian TRIN (Taxonomy Research & Information Network, http://www.taxonomy.org.au/) projects. These efforts support the compilation of large distributed datasets, descriptions and identification of biota. One of the tools developed in association with the EDIT initiative are the Scratchpads (http://scratchpads.eu), a Web 2.0 Virtual Research Environment, that enable taxonomists to collaborate in the production of websites documenting the diversity of life. Using Blagoderov et al. (2010a) as a guide we set up the Australasian Asiloidea Online Scratchpad (http://australasianasiloidea.myspecies.info/). We initially included for public view the published diagnoses of genera and species of the Exoprosopini (Bombyliidae: Anthracinae) and the *Taenogera* genus-group (Therevidae: Agapophytinae). Pages including images, diagnoses, and descriptions were established for each of the undescribed species in the Australasian Asiloidea Online Scratchpad, but hidden from public view until publication.

The paper has been semantically tagged and enhanced using the Pensoft Mark Up Tool (PMT) which is based on the US National Library of Medicine’s DTD (Document Type Definitions) TaxPub extension http://sourceforge.net/projects/taxpub). We intend parallel release of the publication on paper and on-line accompanied by a) links to archived images on Morphbank, and b) with registration of authors, publications, taxon names and other nomenclatural acts in Zoobank, with assignment of Life Science Identifiers (LSIDs) for each new taxa as per the recent proposed amendment to the *International Code of Zoological* nomenclature for a universal register for animal names (Polaszek et al. 2005a; Polaszek et al. 2005b; ICZN 2008). The final XML output of the paper will be archived in PubMedCentral, a PDF uploaded in the Biodiversity Heritage Library (BHL), and all revised species registered in ZooBank (Penev et al. 2010).

### Data resources

The nomenclatural and distributional information will be included in the Australian Faunal Directory (AFD), an open-access online catalogue of taxonomic and biological information on all animal species known to occur within Australia (ABRS, 2009), and the Australian Natural Heritage Assessment Tool (ANHAT), an open-access online map-supported database developed by the Australia Heritage Division of the Department of Sustainability, Environment, Water, Population and Communities that helps identify and prioritise areas for their natural heritage significance, focusing on biodiversity (NHAS 2009). The occurrence data has been uploaded as a Darwin Core Archive (DwC-A), to the Global Biodiversity Information Facility (GBIF) via the Pensoft Data Hosting Center at the GBIF's Integrated Publishing Toolkit (IPT) (http://ipt.pensoft.net/ipt/). The data underpinning the analysis reported in this paper including the data matrix and a most parsimonious tree, together with matrices and trees from Lambkin et al. (2003), were deposited in the Dryad Data Repository (http://datadryad.org/) at http://dx.doi.org/10.5061/dryad.5j64k, the TREEBASE Repository (www.treebase.org/) at http://purl.org/phylo/treebase/phylows/study/TB2:S12050, and at GBIF, the Global Biodiversity Information Facility, http://ipt.pensoft.net/ipt/resource.do?r=bushblitz.

## Phylogenetic analysis

Phylogenetic analysis was based on 207 morphological characters from Lambkin et al. (2003) (Appendix 1). The three new taxa were added to the data matrix from Lambkin et al. (2003) and scored for external morphology including wing venation, and internal morphology of male and female genitalia to produce a matrix for two *Ligyra* outgroup taxa and 40 Australian species belonging to the *Balaana* generic-group in Mesquite version 2.74 (Maddison and Maddison 2010) (Appendix 2 & Appendix 3 LambkinOzBombs2011.nex).

Multistate characters used for phylogenetic analyses have been treated as unordered (non-additive Mickevich and Mitter 1981; Mickevich and Weller 1990). All synapomorphies were weighted equally (Farris 1990). Character polarity was determined by comparison with the outgroups. Variation in morphology between specimens of a taxon was scored as polymorphism and interpreted in the cladistic analyses as “partial uncertainty” (Swofford and Begle 1993) where PAUP* chooses a state from the set of available states that allows minimization of the tree length. There are 66 constant characters in the analysis as the morphological data matrix was based on coding of a much broader taxon sample of 107 worldwide exoprosopine taxa for 207 morphological characters used in Lambkin et al. (2003).

Phylogenetic analyses completed 100 random step-wise addition searches, with tree-bisection-reconnection (TBR) branch swapping, MULPARS, and branches having maximum length zero collapsed to yield polytomies in effect using PAUP*4.0b10 (Swofford 2002).

We used Bremer support (Bremer 1992; Källersjö et al. 1992; Bremer 1994) to measure the strength of evidence for nodes. Bremer support of a group is the difference in length between the tree under consideration and the shortest tree lacking that group. Bremer support values were calculated with TreeRot v.2 (Sorenson 1999) with 20 heuristic searches of the data.

Cladograms and character distribution were analysed in WinClada version 1.00.08 (Nixon 2002) and edited in Adobe Illustrator C S5 version 15.0.2 (Adobe Systems 2010a).

## Results

Cladistic analysis of the 42 taxa of 141 non-constant characters produced five most parsimonious trees (MPTs) of length =931, CI = 0.243, CI excluding uninformative characters = 0.231, and RI = 0.468. The five trees differ only in the placement of *Larrpana dimidiatipennis* (Bowden 1971); as sister to the remaining *Larrpana*, sister to *Muwarna* Lambkin & Yeates 2003, or sister to the *Balaana* genus-group excluding *Wurda* Lambkin & Yeates 2003 and *Kapu* (Lambkin & Yeates 2003). Most parsimonious tree 5 was chosen with reference to the majority-rule consensus tree (Margush and McMorris 1981) as the MPT included those nodes that were found most often in the remaining MPTs. Most parsimonious tree 5 is shown in two parts in Figures 2 and 3 with unambiguous changes on the branches, generic names and Bremer Supports above the branches.

Previous phylogenetic analysis of 207 morphological characters for the worldwide Exoprosopini showed that the Australian bombyliids that were previously placed in *Exoprosopa*, belonged to the monophyletic *Balaana* group of genera, sister to the Australian *Ligyra*. Phylogenetic analysis of characters of the *Balaana* group of genera then led to the description of seven new genera for 42 species in that genus-group (Lambkin et al. 2003). Phylogenetic analysis of the same 207 morphological characters for two *Ligyra* outgroup taxa and 40 Australian species supports the placement of the three new species into existing genera in the *Balaana* generic-group. *Palirika mackenziei* sp. n. and *Palirika culgoafloodplainensis* sp. n. form a clade within the well supported genus, *Palirika* and *Larrpana bushblitz* sp. n. forms a clade with *Larrpana zwicki* within the genus *Larrpana* (Figs 2, 3).

In Lambkin et al. (2003), *Munjua* was erected for three unusual species for which there were few apparent similarities. In that phylogenetic analysis, another particularly aberrant fly, *Munjua trigona* (Fig. 9A, B), was sister to the clade of *Munjua* and the two well-supported terminal clades of *Palirika* and *Balaana* Lambkin & Yeates 2003. This fly clearly did not belong to either *Palirika* or *Balaana* as it possessed none of their diagnostic characters, and was therefore placed in the already heterogeneous *Munjua* rather than creating a monotypic genus (Lambkin et al. 2003).

In this phylogenetic analysis, *Munjua trigona* falls between *Palirika* and *Balaana* as sister to *Palirika* (Figs 2, 3). As this species clearly does not belong to *Palirika*, a new genus *Ngalki* for *Ngalki trigonium* is created.

With the description of the three new species and the transferral of *Munjua trigona* into the new genus *Ngalki*, the three genera *Munjua*, *Palirika*, and *Larrpana* require rediagnoses.

## Taxonomy

### Palirika

Lambkin & Yeates 2003

urn:lsid:catalogueoflife.org:taxon:d916e5f0-29c1-102b-9a4a-00304854f820:col20110201

http://species-id.net/wiki/Palirika

#### Type species:

*Palirika decora*, Lambkin & Yeates, 2003: 812.

#### Rediagnosis.

**Figure 4.**
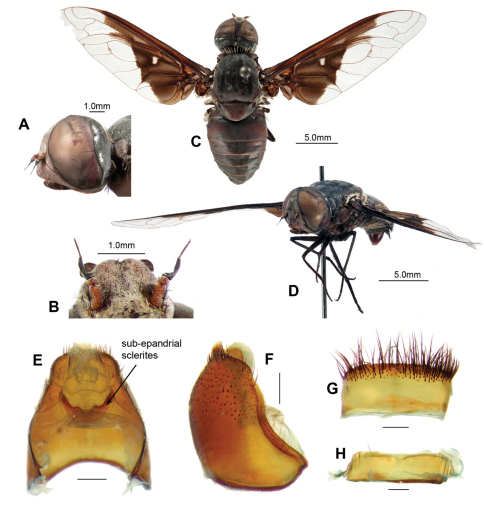
*Palirika culgoafloodplainensis* sp. n., Male holotype. **A** Head lateral **B** Antennae dorsal **C **Adult, dorsal **D** Adult, antero-lateral; Male genitalia: **E** Epandrium ventral with sub-epandrial sclerites **F** Epandrium lateral **G** T_8_, dorsal **H** S_8_, ventral. Scale line (E–H) = 0.5 mm.

**Figure 5.**
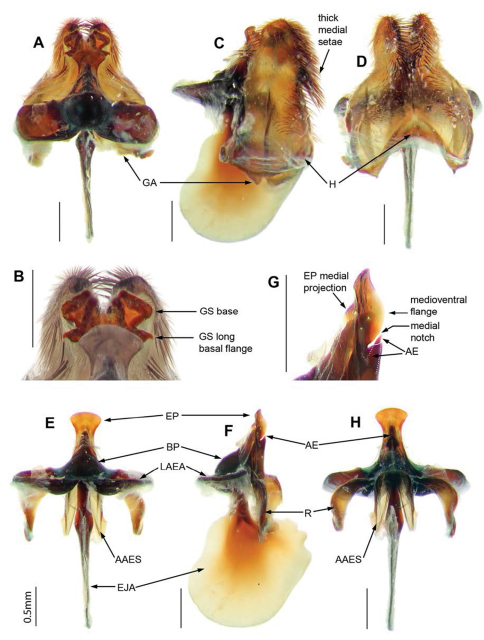
*Palirika culgoafloodplainensis* sp. n., Male holotype genitalia: **A** Gonocoxal complex dorsal **B** Gonocoxal complex lateral **C** Gonocoxal complex ventral **D** Gonostyli **E** Adeagal complex dorsal **F** Adeagal complex lateral **G** Epiphallus lateral **H** Adeagal complex ventral. Scale line = 0.5 mm.

**Figure 6.**
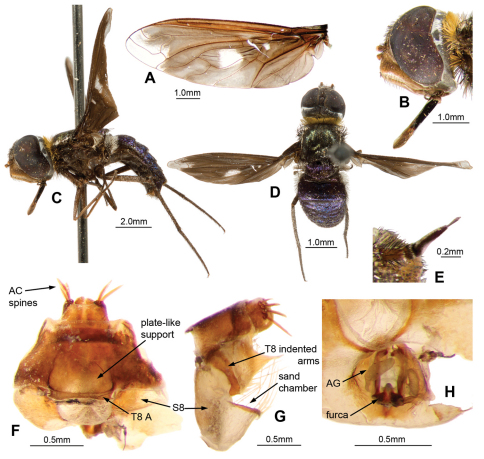
*Palirika mackenziei* sp. n., Female holotype. **A** Wing **B** Head lateral **C** Adult, lateral **D** Adult, dorsal; Male genitalia: **E** Antennae lateral; Genitalia: **F** Dorsal **G** Lateral **H** Dorsal, furca.

Small black, rounded, dense, adpressed metallic scales dorsally on thorax and abdomen (Fig. 6D); no abdominal white scales, sternal vestiture black, not metallic. Epandrium rounded, strongly curved, red, extended smoothly basolaterally (Fig. 4E, F). Gonocoxae deeply narrowed medially, with thickened setae ventromedially, tuft of 6–8 very long, basally-directed, thick setae medially; H projecting in lateral view; EP without lateral lobes, medial projection laterally; LAEA large, convex, extending to G margin; EJA racquet-shaped, longer than the length of G (Fig. 5). Female T_8_ A little more than marginal thickening (Fig. 6F), spermathecal tube more than 8 × length of SP, clear thick-walled ring joining clear thick-walled BB and pigmented subquadrate SR.

Included species: *Palirika anaxios* Lambkin & Yeates 2003, *Palirika basilikos* Lambkin & Yeates 2003, *Palirika blackdownensis* Lambkin & Yeates 2003, *Palirika bouchardi*, *Palirika culgoafloodplainensis* **sp. n.**, *Palirika cyanea* Lambkin & Yeates 2003, *Palirika danielsi* Lambkin & Yeates 2003, *Palirika decora*, *Palirika mackenziei* **sp. n.**, *Palirika marginicollis* (Gray, 1883), *Palirika viridula* Lambkin & Yeates 2003, *Palirika whyalla* Lambkin & Yeates 2003.

### Palirika culgoafloodplainensis

Lambkin sp. n.

urn:lsid:zoobank.org:act:85D57E72-0396-4994-8CF6-7C22B5BAC978

http://species-id.net/wiki/Palirika_culgoafloodplainensis

http://www.morphbank.net/?id=692336

Figs 1C 2 3 4 5 11

#### Material examined.

*Holotype.* **Queensland:** ♂, 28.94°S, 146.918°E, Culgoa Floodplain NP, 7km NNW Toulby Gate, 160m, (CG4AM), Malaise, 20Jan-19Mar2010, C. Kelly, A. Coward, 19273, [dissected], PS1937, A006859, T165704 (QM). Condition: Fair (see remarks below).

#### Diagnosis.

Wing length 20.0 mm

Large dark flies with distinct triangular basal infuscation on the wings wings. Face and frons with transparent scales. Occiput with white scales broadly filling indentation. Collar whitish-cream. Broad laterothoracic stripe of dense white flattened scales. Scutum black with lime-green metallic scales except pink metallic scales anterolaterally to PR bristles and posterolaterally anterior to APA. Scutellum with lime-green metallic scales; very long, white, flattened-scale fringe on posterior margin. Widened base of costa with reddish-brown scales, white scales posteriorly. Wing pattern dimidiate (Fig. 4C); with distinct indentation base of first r_2+3_; extension along R_4+5_, covering basal 1/3 of first r_2+3_ and r_5_; indistinct mottling base of m_1_ along m-m; no infuscated band; anal and posterior cells with apically notched hyaline area, infuscation extending along CuA_2_; cup infuscated basal 4/5; anal infuscated basal 2/3. Squama edged with dense white scales admixed with some reddish-brown scales. T_1_ with Ma white dorsally, black medially and ventrally, dense very long flattened white scales posterolaterally. Abdominal tergites black with bluish-green scales. Epandrium with long setae grouped loosely apically. Epiphallus with short, medial projection. G with long black setae medially, directed basally, longest on weak ventral ridge; LAEA very large, extending well beyond G margins.

#### Description.

*Male. Head* (Figs 4A–D). Face red with transparent scales, frons brown with transparent scales; setae black, frontal depression distinct. Antennal scape red, 3 × length of pedicel, with long black setae dense laterally and ventrally; pedicel red; PP black, conical, 3 × length of pedicel, distinct apical joint; BSM rod-like, black, 3 × length of pedicel; ASM black, conical, length at least width of BSM (Fig. 4B). Occiput with white scales broadly filling indentation (Fig. 4A).

*Thorax.*(Figs 4C–D). Collar whitish-cream. Broad laterothoracic stripe of dense white flattened scales (Fig. 4D). Scutum black with lime-green metallic scales except pink metallic scales anterolaterally to PR bristles and posterolaterally anterior to APA; black setae. Pleural hairs black with reddish-brown iridescence. AN with black Ma; long, lightly iridescent scales at base of wing reddish-brown; long flat broad pale brown scales posteromedially. K with very long fine reddish-brown scales medially. Ma on LT black with reddish-brown iridescence. Tympanal ridge and PL with dense very long fine white flattened scales. Scutellum red, darker basally with lime-green metallic scales; very long, white, flattened-scale fringe on posterior margin. *Legs*. Legs reddish-brown, darkening apically, with black scales and setae, tarsi dark reddish-brown to black; fore-tarsi with straight microtrichia. Pulvilli sharp, curved, 1/3 length of mid- and hind-tarsal claws. Halter knob reddish-brown with apical margin yellow. *Wing* (Fig. 4C), cup narrowly open or closed only at wing margin. Patagium distinct with dense white long flat scales. Widened base of costa with reddish-brown scales, white scales posteriorly. Wing pattern dimidiate (Fig. 4C); with distinct indentation base of first r_2+3_; extension along R_4+5_, covering basal 1/3 of first r_2+3_ and r_5_; indistinct mottling base of m_1_ along m-m; no infuscated band; anal and posterior cells with apically notched hyaline area, infuscation extending along CuA_2_; cup infuscated basal 4/5; anal infuscated basal 2/3. Anal basal edge with dense black scales; alula edged with dense reddish-brown scales; squama edged with dense white scales admixed with some reddish-brown scales.

*Abdomen.*Black, T_1–4_ dark reddish-brown posterolaterally; tergites with bluish-green scales; T_1_ with Ma white dorsally, black medially and ventrally, dense very long flattened white scales posterolaterally; T_2–7_ with tufts of long, black setae laterally and posteriorly. Sternites black with dark reddish-brown scales and hairs. *Genitalia* (Figs 4E–H, 5A–H). Epandrium strongly convex, red with convex apical margin; tapering basal flange; long, black setae loosely grouped apically; SES large, fused medially (see Fig. 4E). Gonocoxae red, narrowed apically; GA short, triangular; thick tufts of long black setae medially, directed basally, longest on weak ventral ridge (Fig. 5C); EJA very large, extending well beyond gonocoxal margins, racquet-shaped; LAEA very large, extending well beyond G margins, deeply convex (Fig. 5A); AAES strong wedges (Fig. 5E, H); GS (Fig. 5B) cupped within G margins, large subquadrate base projecting apically; EP long, expanded slightly apically, without lateral lobes, short medial projection; medioventral flange above AE present (Fig. 5G); large recurved R (Fig. 5F, H); H triangular, projecting slightly in lateral view (Fig. 5C).

*Female.* Unknown.

**Etymology.** This species is named *culgoafloodplainensis* after the remote Queensland Culgoa Floodplain National Park where the type specimen was collected, and where CLL and Noel Starick received so much hospitality, enthusiasm, and encouragement over the years from all the staff, but especially RIC Andy Coward.

#### Distribution.

(Fig. 11). This species has only been collected from the type locality in central south-western Queensland.

#### Remarks.

Due to extended storage in propylene glycol as retrieval of sample was prevented by extensive and prolonged flooding the specimen bears few setae, hairs or scales, therefore colour patterns referred to in the description are based on those remaining, usually at junctions of sclerites.

### Palirika mackenziei

Lambkin sp. n.

urn:lsid:zoobank.org:act:CD50600C-901E-46CA-840F-D4FEA863AF0E

http://species-id.net/wiki/Palirika_mackenziei

http://www.morphbank.net/?id=692335

Figs 1A–B 2 3 6 11

#### Material examined. 

*Holotype.* **Queensland:** ♀, 26°43.7'S, 142°39.1'E, Plevna Downs, Tompilly Hill summit, 13 Dec 2007, C. Lambkin, N. Starick & R. Mackenzie, 15454, sweep net, hilltopping, 220m, [dissected], PS1893, A007533, T152481 (QM). Condition: Good.

#### Diagnosis.

Wing length 9.0 mm.

Small dark flies with heavily infuscated wings, hyaline only apically and medial spot. Face orange with shiny reddish-brown scales, frons black with shiny black scales. Collar yellow. Narrow laterothoracic stripe of whitish scales. Scutum black with dull lime-green metallic scales except pinkish metallic scales anterolaterally and posteromedially. Scutellum dark brown, darker basally with royal-blue metallic scales, purple metallic scales laterally and posteriorly. Widened base of costa with shiny reddish-brown scales, no paler scales posteriorly. Wing pattern broadly dimidiate (Fig. 6A); black with hyaline areas, apically and medially. Apical hyaline area covering extreme apex of r_1_, apex of first r_2+3_, apical half of second r_2+3_, all r_4_, and extreme apex r_5_. Medial hyaline area covering middle of dc extending from M_1_ across m-cu and into m_2_. Paler prediscoidal opaque area distinct. Alula and squama edged with long broad grey scales. T_1_ with white Ma; long white flattened scales posterolaterally. Tergites black with royal-blue metallic scales that reflect purple (Fig. 6D). Female T_8_ A short, plate-like support distinct.

#### Description.

*Female. Head.*(Figs 6B–E). Face orange with shiny reddish-brown scales, frons black with shiny black scales; setae black, longest below distinct frontal depression. Antennal scape red, 3 × length of pedicel, with long black setae dense laterally and ventrally; pedicel red with black setae shorter and sparser dorsally; PP conical, 5 × length of pedicel, black with silvery pruinescence, distinct apical joint; BSM rod-like, expanded apically, reddish-brown, 2 × length of pedicel; ASM reddish, conical, length less than width of BSM (Fig. 6E). Occiput with shiny black scales broadly filling indentation.

*Thorax.* (Figs 6B–D). Collar yellow. Narrow laterothoracic stripe of whitish scales. Long white flattened scales posteromedially. Scutum black with dull lime-green metallic scales except pinkish metallic scales anterolaterally and posteromedially; black setae. AN with black Ma, long yellow setae anteriorly; long, lightly iridescent scales at base of wing black. Prealar bristles strong, black and long. Postalar bristles strong, black and reaching almost apex of scutellum. Pleural hairs black with reddish iridescence. Ma of LT black, reddish-brown dorsally. Tympanal ridge and PL with yellowish flattened scales. Scutellum dark brown, darker basally with royal-blue metallic scales, purple metallic scales laterally and posteriorly; strong, black apical bristles. *Legs*. Reddish-brown, tarsi darker; scales and setae black. Pulvilli sharp, curved, half length of mid- and hind-tarsal claws. Halter knob dark reddish-brown, posteromedial edge broadly yellow. *Wing* (Fig. 6A), cup open. Widened base of costa with shiny reddish-brown scales, no paler scales posteriorly. Wing pattern broadly dimidiate; black with hyaline areas, apically and medially. Apical hyaline area covering extreme apex of r_1_, apex of first r_2+3_, apical half of second r_2+3_, all r_4_, and extreme apex r_5_. Medial hyaline area covering middle of dc extending from M_1_ across m-cu and into m_2_. Paler prediscoidal opaque area distinct. Anal edged with long black scales basally. Alula edged with long broad grey scales. Squama edged with dense overlapping long grey scales.

*Abdomen.* (Figs 6C–D). T_1_ with white Ma; long white flattened scales posterolaterally. Tergites black with royal-blue metallic scales that reflect purple when not viewed dorsally, pleura with lateral tufts of long black setae on T_2–7_. Sternites black, with black scales and setae. *Genitalia* (Fig. 6F–H). Dorsal T_8_ A short, plate-like support distinct; T_10_ with 4 pairs of stout AC spines. Furca with 2 long broad posteriorly directed arms with small recurved hook-like dorsal extensions apically.

*Male.* Unknown.

#### Etymology.

This species is named *mackenziei* to acknowledge the enthusiasm and interest in all kinds of natural history by the Mackenzie family of Plevna Downs Station where the type, and only, specimen was collected. Since 2007, following the discovery of dinosaurs on their property, together with a large undescribed spider, CLL and Noel Starick have been welcomed by the Mackenzie family. Robyn Mackenzie was thrilled to be helping catch hill-topping beeflies on the summit of Tompilly Hill when the only female specimen of this unusual *Palirika* was captured (Fig. 1A). We have happily instructed the family, the local Natural History Society, students, teachers, regional property owners and community members on the ins and outs of biodiversity of arid areas, especially the insects.

#### Distribution.

(Fig. 11). This species has only been collected from the type locality in remote far south-western Queensland.

### Larrpana

Lambkin & Yeates 2003

urn:lsid:catalogueoflife.org:taxon:d9155668-29c1-102b-9a4a-00304854f820:col20110201

http://species-id.net/wiki/Larrpana

#### Type species:

*Exoprosopa dimidiatipennis* Bowden 1971: 64.

#### Rediagnosis.

**Figure 7.**
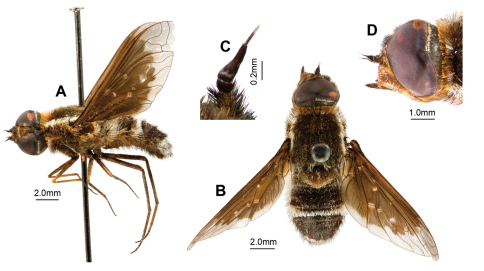
*Larrpana bushblitz* sp. n. **A** Adult, lateral **B** Adult, dorsal **C** Antennae dorsal **D** Head lateral.

Dimidiate wing pattern as in Figure 7A–B, dark basally, hyaline apically; infuscation forming a distinctly separated, basal triangle leaving the apex of the posterior cubital and anal cells broadly hyaline. Cream laterothoracic stripes, white scale bands on T_3_, sparse white scales on T_6–7_. Epandrial basolateral flange longer and broader than the length of the epandrial base. Gonocoxae deeply narrowed medially, tufts of thickened setae on distinct ventral flange that projects basally; EP with rounded, projecting, lateral lobes; short, wedge-shaped AAES; EJA long. Sperm pump long with unpigmented papillae, collar or clear ring surrounding join between BB and thick-walled round SR.

Included species: *Larrpana bushblitz* sp. n., *Larrpana dimidiatipennis*, *Larrpana collessi* Lambkin & Yeates 2003, *Larrpana zwicki*.

### Larrpana bushblitz

Lambkin sp. n.

urn:lsid:zoobank.org:act:1CFF61E9-10C6-4185-8135-CDA4DAEB69A9

http://species-id.net/wiki/Larrpana_bushblitz

http://www.morphbank.net/?id=692334

Figs 1D 2 3 7 8 11

#### Material examined. 

*Holotype.* **Western Australia**: ♂, 29.302°S, 116.725°E, Karara, 23.5km ESE Boiada Camp, 356m, 17 Sep 2009, Lambkin, sweeping, 18402, rocky hilltop, hilltopping, PS1714, WAM 82396 (WAM). Condition: Good.

Paratypes. *Western Australia:* 1♂, same data as holotype, T152479 (QM); 1♂, 29.309°S, 116.731°E, Karara, Forest lookout, 24.4km SE Boiada Camp, 17 Sep 2009, 18405, Lambkin, sweeping, 410m, rocky hilltop, hilltopping, [dissected], PS1894, WAM 82397 (WAM); 1♂, same data Forest lookout, A007534, T152480 (QM).

#### Diagnosis.

Wing length 14 mm

**Figure 8.**
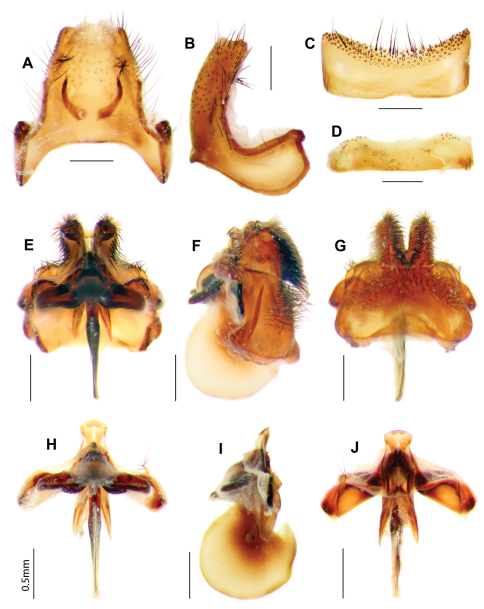
*Larrpana bushblitz* sp. n. Male genitalia: A) Epandrium ventral with sub-epandrial sclerites **B **Epandrium lateral **C** T_8_, dorsal **D** S_8_, ventral; Male genitalia: **E** Gonocoxal complex dorsal **F **Gonocoxal complex lateral **G** Gonocoxal complex ventral **H** Adeagal complex dorsal **I** Adeagal complex lateral **J **Adeagal complex ventral. Scale line = 0.5 mm.

Medium, dark, densely setose, flies with black, dimidiate wings with five indistinct yellowish spots (Fig. 7A, B); infuscation indented in 1st r_2+3_; no lobe or medial band; dc infuscated except for rectangular hyaline area at junction of m-cu and m-m. Thoracic collar yellow. Dorsal surface of thorax, scutellum and abdomen covered with long upstanding setae, producing distinct fluffy appearance. T_3_ with uninterrupted white band of upstanding scales narrowing medially, laterally spanning entire tergite. Alula and squama edged with dense long cream scales; proximal 1/3 of anal cell edged with black scales, longest basally. Male (Fig. 8) E with basal flange very long, broad, extending basally, apically recurved. H large, subquadrate in lateral view, distinctly projecting.

#### Description. 

*Male. Head*(Fig. 7A–D). Frons reddish-brown, face red, face and frons with transparent scales, black setae longest below shallow frontal depression. Antenna (Fig. 7C). Scape 2.5 × length of pedicel, red; pedicel red; PP long, 3–4 × length of pedicel, as long as scape and pedicel combined, dark reddish-brown, with reddish pruinescence; BSM dark reddish-brown, 2 × length of pedicel, not expanded apically; ASM minute blunt cone, length less than width of BSM. Narrow band of cream scales at posterior margin of eye medially.

*Thorax* (Fig. 7A, B). Collar yellow. Very broad distinct laterothoracic stripe of dense long white scales. Scutum black; scales long, reddish-brown, white posteriorly; long dense black setae, longest anteriorly and posteriorly. AN and PN with Ma admixed black and reddish-brown; long, slightly iridescent, reddish-brown scales at base of wing. Pleural hairs black, with reddish-brown iridescence. Scales on APA white. Laterotergite with dark reddish-brown Ma ventrally, white dorsally and red medially. Plumula with dense long white scales and TR with dense long yellow scales. Scutellum dark reddish-brown, black basally; scales black basally, transparent pale-brown medially and posteriorly, posterior scales longest; long dense, black setae. Legs reddish-yellow with black scales. Microchaetae on fore-tarsi curved apically. Pulvilli straight sharp cones, more than 1/3 length of mid- and hind-tarsal claws. Halter knob red with pale whitish apical edge. *Wing* (Fig. 7A, B). Widened base of C with black scales with pale band posteriorly. Spur-veins present on base of R_4_ extending into r_4_ and on apex of m-cu extending into m_2_ in some specimens; bump at basal bend of m-cu. Wing pattern (Fig. 7A, B) black, dimidiate, broad basal infuscation following R proximal to i-r_1_ to wing margin in apical 4/5 anal cell; indented in 1st r_2+3_; no lobe or medial band; dc infuscated except for rectangular hyaline area at junction of m-cu and m-m; apex hyaline. Dark yellowish-brown areas bordering base of CuA_1_, join of R_1_ and R_s_, r-m continuing onto base of R_2+3_, and base m-cu; together with prediscoidal opaque area forming indistinct pentagonal pattern of spots within infuscation. Anal and cup infuscated for basal 4/5. Alula and squama edged with dense long cream scales; proximal 1/3 of anal cell edged with black scales, longest basally.

*Abdomen.* (Fig. 7A, B). Tergites black with red anterolateral areas medially rounded on T_2–3_ < 1/4 width of tergite. Scales dense, black except: T_3_ with uninterrupted white band of upstanding scales narrowing medially, laterally spanning entire tergite; T_1–3_ with dense long white upstanding lateral scales; T_6–7_ with white scales. T_1_ with Ma white dorsally and laterally, yellow ventrally. T_1–3_ with long dense white setae laterally, pale brown anteriorly, and black posteromedially; T_4–7_ with long dense thick black setae. Sternites red with sparse long pale reddish scales, dense long black setae. *Genitalia* (Fig. 8). Epandrium red with distinct anterolateral flange bearing cluster of long black setae; basal flange very large, long, broad, extending basally and apically upcurved; setae black, loosely grouped anterolaterally; SES very long, linear, broadened basally. Gonocoxae red, strongly narrowed medially; ventral ridge projecting; LAEA deeply convex; GS cupped within G margins, subquadrate base projecting apically; very large recurved rami extending beyond G margins; setae long black, not short apically, dense tufts of long, thickened setae medially, directed basally, very long thin setae continuing laterally; H large, subquadrate in lateral view, distinctly projecting. Epiphallus 1.4 × neck width; with apical margins inturned forming projecting rounded lobes.

*Female.* Unknown.

#### Etymology.

This species is named as a noun in apposition after the three-year, multimillion dollar Bush Blitz program that organised and funded the survey on Charles Darwin Reserve, Karara, Lochada and Kadji Kadji Pastoral Leases in Western Australia on which this species was collected. The core focus of the Bush Blitz program is to document the plants and animals in hundreds of properties across Australia’s National Reserve System, and on nature discovery – identifying and describing new species of plants and animals. The Bush Blitz program also funded the survey in western New South Wales and Queensland on which *Palirika culgoafloodplainensis* sp. n. was collected (ABRS BB 2009/23887) and funded the description of these three species (ABRS BB TTG209-06).

#### Distribution.

(Fig. 11). *Larrpana bushblitz* sp. n. has only been collected from Karara Pastoral Lease, 213 km ESE of Geraldton in Western Australia.

#### Comments.

On collection, this species appeared similar to the two male specimens of *Larrpana zwicki* collected only near Windorah (Lambkin et al. 2003) and phylogenetic analysis (Figs 2–3) indicates a close relationship between the two.

### Munjua

Lambkin & Yeates 2003

urn:lsid:catalogueoflife.org:taxon:d916e848-29c1-102b-9a4a-00304854f820:col20110201

http://species-id.net/wiki/Munjua

#### Type species:

*Munjua erugata* Lambkin & Yeates, 2003: 795.

#### Rediagnosis.

Wing with medial hyaline band not linear and narrowing apically, apical infuscated band not meeting posterior wing margin more broadly than medial hyaline band. Gonocoxae deeply narrowed medially, broadly indented basally, with tufts of thickened setae ventromedially, H projecting but not forming a finger-like extension; AE short; EP with medioventral process above AE; very long AAES reaching G margins; EJA racquet-shaped, very long. Sperm pump short with unpigmented papillae, apical endplate simple with thin processes; thick-walled round SR with distinct basal bulb.

Included species: *Munjua erugata* Lambkin & Yeates 2003, *Munjua lepidokingi* Lambkin & Yeates 2003, *Munjua paralutea* Lambkin & Paramonov 2003.

#### Comments.

See reference to the rediagnosis of the genus *Munjua* in the phylogenetic results.

### Ngalki

Lambkin gen. n.

urn:lsid:zoobank.org:act:77AACA67-1FA6-4CD9-871E-D95C34FDE977

http://species-id.net/wiki/Ngalki

#### Type species:

*Munjua trigona* Lambkin & Yeates, 2003: 804.

#### Diagnosis.

**Figure 9.**
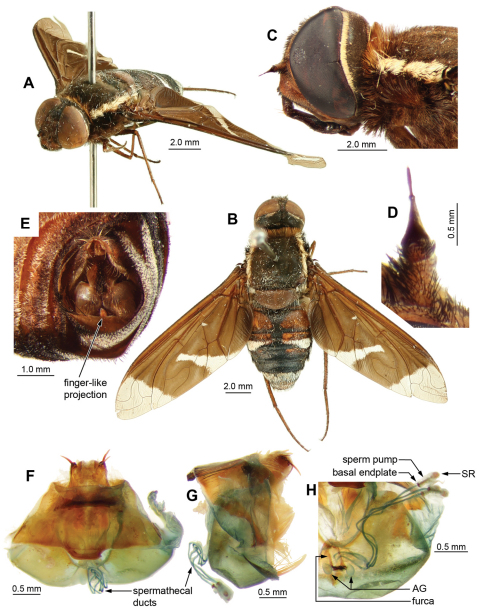
*Ngalki trigonium*. **A** Adult, antero-lateral **B** Adult, dorsal **C** Head and thorax lateral **D **Antennae lateral **E** Male genitalic complex showing diagnostic finger-like projection on hypandrium, clearly visible *in situ*. Female genitalia: **F** Dorsal **G** Lateral **H** Dorsal, furca and spermathecal complex.

**Figure 10.**
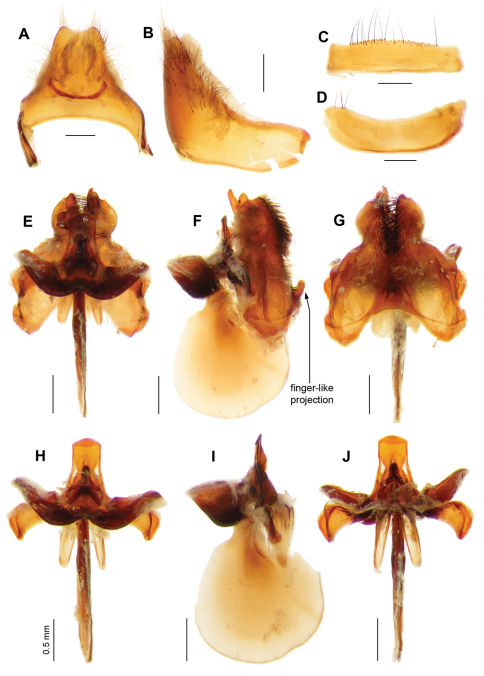
*Ngalki trigonium*. Male genitalia: **A** Epandrium ventral with sub-epandrial sclerites **B **Epandrium lateral **C** T_8_, dorsal **D** S_8_, ventral **E** Gonocoxal complex dorsal **F** Gonocoxal complex lateral showing diagnostic finger-like projection on hypandrium **G** Gonocoxal complex ventral **H** Adeagal complex dorsal **I** Adeagal complex lateral **J** Adeagal complex ventral. Scale line = 0.5 mm.

Wing with medial hyaline band linear and narrowing apically, apical infuscated band meeting posterior wing margin twice breadth of medial hyaline band (Fig. 9A, B). Gonocoxae deeply narrowed medially, broadly indented basally, with tufts of thickened setae ventromedially, H projecting forming finger-like extension (Figs 9E, 10F); AE short; EP with medioventral process above AE; very long AAES reaching G margins; EJA racquet-shaped, very long (Fig. 10). Sperm pump short with unpigmented papillae, apical endplate simple with thin processes; thick-walled round SR with no basal bulb (Fig. 9F–H).

#### Etymology.

The name for the genus *Ngalki* is from the aboriginal term *ngalki* for “little finger” from the Ngiyampaa language spoken in much of central New South Wales (Donaldson 1994), referring to the diagnostic character of the male genitalia for this genus, and is treated as neutral. This follows the tradition set in Lambkin et al. (2003), of using appropriate aboriginal terms for the names of new genera of Australian exoprosopines.

Included species: *Munjua trigona* Lambkin & Yeates, 2003

#### Comments.

See reference to the erection of the genus *Ngalki* in the phylogenetic results.

### Ngalki trigonium

(Lambkin & Yeates) comb. n.

urn:lsid:catalogueoflife.org:taxon:db706730-2dc5-11e0-98c6-2ce70255a436:col20110201

http://species-id.net/wiki/Ngalki_trigonium

http://www.morphbank.net/?id=692333

Figs 2 3 9 10

Munjua trigona Lambkin & Yeates, 2003: 804.

#### Material examined.

*Paratypes.* **New South Wales:** 1♂, Round Hill Nature Reserve, 27 Dec 1976, G. Daniels, GDCB Reg # 14199, K 253709; 1♀, Round Hill Nature Reserve, same, GDCB Reg # 17925, K 253717 (AM). **Victoria:** 1♀, Wyperfield Nat Park, 7 Dec 1976, G. Daniels, GDCB Reg # 14163, K 253707; 3♂, Wyperfield Nat Park, 8 Dec 1976, G. Daniels, GDCB Reg # 17924, #14164, # 17923, K 253720, (PS1936) K 253702, K 253712 (AM).

#### Other material.

**New South Wales:** 1♀, Round Hill area, 24–25 Nov 1991, A. Sundholm, [dissected], PS1935, K 289927 (AM). **Western Australia:** 1♂, Fraser Range, 8 Nov 1977, A. Atkins, [dissected], GDCB Reg # 14165, PS1934, K 253698 (AM).

#### Rediagnosis.

Large dark flies (wing length 15-20 mm), wings as in Figure 9B, dark with narrow, linear, medial hyaline band; long, finger-like apically-directed projection on hypandrium (Figs 9E, 10F). Laterothoracic stripe creamy-white. Broad white scale band on T_3_, T_4–5_ with black scales, T_6–7_ with white scales. Epandrium with long golden setae; SES joined medially. Epiphallus with short medial projection. Female with no BB between pump and pale, square SR.

#### Redescription. 

*Male. Head* (Fig. 9C). Face and frons red with reddish-yellow scales, black setae longest below deep frontal depression. Antennal scape 3 × length of pedicel, red; pedicel red; PP 3.5 × length of pedicel, black with reddish pruinescence; BSM dark reddish-brown, 3.5 × length of pedicel, expanded apically; ASM short, blunt (Fig. 9D). Narrow line of creamy-white scales on posterior margin of eye.

*Thorax.* Collar yellow, with tips of Ma darker, reddish. Laterothoracic stripe broad creamy-white, distinct. Scutum black with long hair-like reddish-brown scales, sparse white flattened scales posteromedially. Pleural vestiture dark-red with mauve iridescence; admixed dark-red and black Ma on PE and AN; long scales at base of wing with mauve iridescence. Anepimeral setae admixed dark-red and black. Scales on APA yellow. Plumula and TR hairs creamy yellow. LT with black Ma, red dorsally. Scutellum red with scales yellowish-red, setae black, sparse long white scale fringe on posterior margin. Legs red with dark reddish-brown scales. Pulvilli chisel-like wedges, less than a half length of mid- and hind-tarsal claws. Halter knob dark reddish-brown, with yellow apical edge. *Wing* (Fig. 9B). R_4+5_ with abrupt bend basally, cup closed at wing margin, or narrowly open. Widened base of C with dark, reddish-brown scales, paler brown scales posteriorly. M_2_ very sinuous, width of m_1_ at wing margin < 1/2 width of m_2_. Wing pattern (Fig. 9A, B) black, with broad infuscated band following R proximal to i-r_1_, obliquely through 1st r_2+3_, r_5_ and m_1_ to meet wing margin in m_2_; no infuscation in 2nd r_2+3_. Hyaline band very narrow, linear, from dc through m_2_ basally, apex of cua, cup and anal cells. Squama with reddish-yellow scales.

*Abdomen* (Fig. 9B). Integument with large red areas laterally, leaving black medial band; T_2_ with black medial band broad basally, width > 1/3 width of the tergite, tapering sharply apically; T_3–4_ with black medial band < 1/4 width, black apical band; T_5–7_ with red areas laterally < 1/4 width. T_1_ with Ma white, reddish-brown scales medially. T_2_ with dense long cream hairs anterolaterally, dark reddish-brown scales, some white scales laterally. Broad white scale band on T_3_, interrupted medially, dark reddish-brown scales anteromedially, black scales posteromedially. T_4–5_ with black scales, T_6–7_ with white scales. Sternites red; S_2–3_ and S_4–5_ basally with dense white scales and dense long, fine white hairs; S_4–5_ apically with dark reddish-brown scales, S_6–7_ with black scales, black setae. *Genitalia* (Figs 9E, 10). Epandrium red with basal flange short and broad; setae black, long golden setae apically; SES joined medially. Gonocoxae red, strongly narrowed medially; setae black, short apically, medially with thick tufts of basally directed setae, long setae laterally around apex of H; distinct ventral ridge; LAEA deeply convex, extending past G margins; GS cupped within G margins, large subquadrate base with slight projection apically; large recurved R; H crescent-shaped, laterally delimited by swollen and expanded G, laterally subrectangular, with distinct large, blunt finger-like apical projection. Epiphallus long, not expanded apically; with medial projection.

*Female.* Same as male. *Genitalia* (Fig. 9F–H). Dorsal T_8_ A short, entire; T_10_ with 4 pairs of short, thick AC spines; apical endplate with long thin processes; basal endplate with thick processes; long unpigmented papillae, no BB, narrow ring between pump and lightly pigmented, square SR.

**Figure 11.**
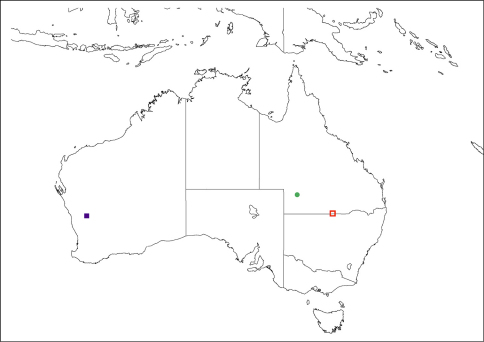
Map of distribution. Closed circle - *Palirika mackenziei* sp. n. Open square - *Palirika culgoafloodplainensis* sp. n. Closed square - *Larrpana bushblitz* sp. n.

#### Etymology.

In Lambkin et al. (2003), the name *trigona* given to this species was derived from the Greek *trigonas* “triangular” This was the name the late Sergei Paramonov gave to this species in his unpublished manuscript, and was used to honour his extensive work on Australian bombyliids. With the transfer to *Ngalki*, the specific emendation requires adjustment, and becomes *trigonium* to reflect the neutral gender of the new genus-group name.

#### Distribution.

This species has been collected in the southern Australian Bassian region, from semi-arid and arid mallee areas.

#### Comments.

The finger-like projection on the H (Figs 9E, 10F) in the males of *Ngalki trigonium* is apparent without dissection and, together with the unusual wing pattern, allows easy identification.

## Keys

### Key to the Genera of the Australian *Balaana* Group

**Table:** 

1	Metallic scales on body, black reflecting blue, bluish-black, green or maroon, no white or yellow scales on T_2–7_ or on S_2–7_ (Fig. 6D)	*Palirika* Lambkin & Yeates
–	No metallic reflecting black scales; white or yellow scales present T_2–7_, usually distinct bands or lateral triangles on T_3_ (Fig. 9B)	2
2 (1)	Wing dimidiate and at least apical half of anal cell margin hyaline, at most short narrow lobe following m-m into m_2,_ no medial hyaline band (Fig. 7B)	*Larrpana* Lambkin & Yeates
–	Wing not dimidiate or anal cell fully infuscated; distinct medial hyaline band usually present (Fig. 9B)	3
3 (2)	Female spermathecal reservoir a long cylinder; T_2_ with black scales; T_6–7_ with white scales; proboscis extending beyond oral cavity, not longer than head	*Balaana* Lambkin & Yeates
–	Female spermathecal reservoir round to subquadrate never a long cylinder; T_2_ with some yellow scales unless T_6_ or T_7_ with black scales or proboscis longer than head ..	4
4 (3)	Male with no medioventral process on epiphallus above aedeagus, anterior arms of aedeagal sheath long, reaching gonocoxal margins; quadrate sub-epandrial sclerites in epandrium. EITHER Deeply infuscated wings, only apex hyaline; paler yellowish spots at base of R_2+3_, at base of CuA_1_, join of R_1_ and R_s_, r-m and base of m-cu; T_6_ black scales; OR medial hyaline band a narrow line; black scales forming median circle apex of T_2_ and base of T_3_, yellow scales anteriorly and laterally on T_2_, medially and laterally on T_3_	*Muwarna* Lambkin & Yeates
–	Male with medioventral process on epiphallus above aedeagus (Figs 5G, 10I), linear or single fused (Fig. 10A) sub-epandrial sclerites in epandrium. Wings less infuscated with broad medial hyaline band broader posteriorly; IF deeply infuscated with medial hyaline band a narrow line (Fig. 9B) no median circle of black scales on T_2–3_	5
5 (4)	Male with anterior arms of aedeagal sheath long, reaching gonocoxal margins (Fig. 10E). Ventral ridge on gonocoxae small or absent, not projecting basally AND hypandrium projecting. Epiphallus without lateral lobes; ejaculatory apodeme extending beyond gonocoxae by more than length of gonostylus (Fig. 10E). EITHER yellow vestiture with wing infuscation distinctly variegated, bright yellow basally and medial band dark brown to black; OR no yellow scales on T_2–7_ (Fig. 9B); OR only yellow scales anteromedially on T_2–3_, S_2–3_ with dense, white scales and setae, S_5–7_ with dense, black scales and setae	6
–	Male with anterior arms of aedeagal sheath short, not reaching gonocoxal margins; if ventral ridge on gonocoxae very small or absent then hypandrium not projecting. Abdominal yellow scales at least anteriorly T_2_, S_2–3_ with dense, white scales; hemispherical tufts of macrochaetae laterally on T_1_ white or yellow, not dark reddish-brown or black; wing infuscation not distinctly variegated, IF yellow scales only anteromedially T_2_ then scales on S_5–7_ not black, at most reddish-brown	7
6 (5)	Medial hyaline band linear, narrowing anteriorly, with apex of anal cell and cup hyaline (Fig. 9B); male gonocoxae with long, finger-like projection from hypandrium (Figs 9E, 10F)	*Ngalki* Lambkin gen. n.
–	Medial hyaline band not linear, not narrowing anteriorly; male gonocoxae with no finger-like projection from hypandrium	*Munjua* Lambkin & Yeates
7 (5)	Male epandrium with strongly grouped setae on anterolateral flange; ventral ridge on gonocoxae large, distinctly projecting basally, hypandrium not projecting, epiphallus with rounded projecting lateral lobes, ejaculatory apodeme short, extending beyond gonocoxae by less than length of gonostylus, hind-tibial scales not protruding, dark flies, T_2_ and T_4_ mostly black scales	*Kapu* (Lambkin & Yeates)
–	Male epandrium with loose setae, without an anterolateral flange; hind-tibial scales protruding, pale yellowish flies with striped abdominal vestiture, T_2_ and T_4_ mostly yellow scales	*Wurda* Lambkin & Yeates

### Key to Species of Palirika

**Table:** 

1	No pre-apical infuscated band on wing (Figs 4C, 6A)	2
–	Pre-apical infuscated band on wing present	3
2 (1)	Infuscation of wing blade almost complete except for hyaline apical area and isolated spot over dc (Fig. 6A)	*Palirika mackenziei* Lambkin sp. n.
–	Infuscation of wing blade only extending over half wing area, indistinct extension along R_4+5_ and isolated mottled area along m-m (Fig. 4C)	*Palirika culgoafloodplainensis* Lambkin sp. n.
3 (1)	Anal and posterior cells with notched hyaline area, infuscation extending along CuA_2_; apically-directed spur-vein on i-r_1_ cross vein	*Palirika bouchardi*
–	Anal and posterior cells without extension along CuA_2,_ rarely spur-vein on i-r_1_ cross vein	4
4 (3)	Hyaline medial band continues anteriorly through entire r_5_; dark thorax; abdomen: males dark prussian-blue, almost black; females bluish-green	*Palirika cyanea*
–	Hyaline band usually through dc anteriorly, not entirely through r_5_, or absent; thorax and abdomen not as above	5
5 (4)	Collar white with contrasting tuft of black lateral Ma at base of pronotal lobe, bright green thorax, anterolaterally dark maroon; bright, dark blue abdomen; anal and cup fully infuscated	*Palirika decora*
–	Collar entirely white or yellow, at most 4 reddish Ma above postpronotal lobe; thorax, abdomen, anal and cup not as above	6
6 (5)	Face yellow, most facial setae shiny gold; blue-green thorax and abdomen	*Palirika viridula*
–	Face orange-red to reddish-brown; if yellow, most facial setae black; thorax and abdomen not as above	7
7 (6)	Thorax green	8
–	Thorax dark, not green	11
8 (7)	Thorax anterolaterally with dark maroon scales; anal and cup infuscation various	9
–	Thorax entirely green; anal and cup hyaline apically	10
9 (8)	Abdomen bluish-green; brown wing infuscation, anal and cup hyaline apically; blue face scales	*Palirika whyalla*
–	Abdomen entirely purple; black wing infuscation, anal and cup fully infuscated; purple face scales	*Palirika anaxios*
10 (8)	Thorax bright yellowish-green; abdomen blue to bluish-green metallic scales; infuscated wing band short, much narrower than hyaline band	*Palirika marginicollis*
–	Thorax dark bluish-green; abdomen T_2_ blue-green, T_3–7_ blue, T_4–6_ admixed maroon at least laterally; infuscated wing band broader than hyaline band	*Palirika blackdownensis*
11 (7)	Abdomen purple with blue scales on T_4–6_, Queensland	*Palirika danielsi*
–	Abdomen entirely purple, no blue scales on T_4–6_, Western Australia	*Palirika basilikos*

### Key to Species of Larrpana

**Table:** 

1	No yellow scales on T_2–7_ (Fig. 7A, B), Ma on T_1_ black, white or yellow	2
–	Abdominal yellow scales at least anteriorly T_2_; Ma on T_1_ white, not black	*Larrpana collessi*
2 (1)	Wing without small paler yellowish spots in infuscation, m-m without infuscation	*Larrpana dimidiatipennis*
–	Wing with small paler yellowish spots in infuscation; m-m with infuscation (Fig. 7A, B)	3
3 (2)	Wing without short narrow lobe following m-m into m_2_ (Fig. 7A, B)	*Larrpana bushblitz* Lambkin sp. n.
–	Wing with short narrow lobe following m-m in m_1_ into m_2_	*Larrpana zwicki*

## Supplementary Material

XML Treatment for Palirika

XML Treatment for Palirika culgoafloodplainensis

XML Treatment for Palirika mackenziei

XML Treatment for Larrpana

XML Treatment for Larrpana bushblitz

XML Treatment for Munjua

XML Treatment for Ngalki

XML Treatment for Ngalki trigonium

## Appendix 1

### Morphological Characters

For a full description of these characters see Appendix 2 in Lambkin et al. (2003).

Morphological terminology follows that of McAlpine (1981), Yeates (1994), and Lambkin et al. (2003).

### HeadFrontal

1. Head scales: (0) absent; (1) present

2. Face scale density: (0) absent; (1) sparse; (2) overlapping; (3) carpet

3. Frons scale density: (0) absent; (1) sparse; (2) overlapping; (3) carpet; (4) distinct dense medial patch

4. Frons with vertical groove: (0) absent; (1) depression; (2) groove

5. Frons with horizontal depression: (0) absent; (1) shallow; (2) distinct; (3) deep

### Dorsal

6. W head/W thorax: (0) <; (1) =; (2) >

7. L antennae to compound eye/L scape: (0) <; (1) ≥; (2) ≥ 2x; (3) ≥ 4x

8. L antennal separation/L scape: (0) <; (1) ≥; (2) ≥ 2x; (3) ≥ 3x

9. Male compound eye separation /W OT: (0) meet; (1) <; (2) =; (3) ≤ 2x; (4) ≤ 3x; (5) > 3x

10. Female compound eye separation /W OT: (0) ≤ 2x; (1) ≤ 3x; (2) > 3x

11. OT to posterior margin of compound eye/ L OT: (0) ≤ OT; (1) > OT; (2) ≥ 2x; (3) ≥ 3x

12. L occiput/L OT: (0) ≤ 2x; (1) < 3x; (2) occiput long, well developed ≥ 3x

13. L vertex; i.e. L OT to occipital groove/ L OT: (0) ≤ L OT; (1) ≤ 2x; (2) wide > 2x

14. Depth occipital foveal depression: (0) no vertex; (1) not depressed; (2) shallow; (3) deep, slopes posteriorly at 45° to a short OG

15. W occipital foveal depression: (0) no vertex; (1) not depressed; (2) narrower than compound eye separation; (3) wider than compound eye separation

16. Apical occipital groove: (0) narrow; (1) wide rounded

### Lateral

17. Shape of head laterally: (0) round; (1) protruding but rounded, blunt; (2) conical

18. L proboscis: (0) < L oral cavity; (1) > L oral cavity; (2) > L head; (3) > 1.5x L head; (4) > 2x L head

19. L palps/ L proboscis: (0) rudimentary; (1) < 0.25x, short; (2) < 1, long

20. Genae setae surrounding oral cavity: (0) not grouped; (1) grouped laterally; (2) grouped apically, small tuft

21. L setae below antennae/L scape: (0) >; (1) ≤; (2) ≤ 0.5

22. Horizontal depression between antennae: (0) absent or shallow; (1) distinct; (2) deep

23. W of face projection/W compound eye including indentation posterior margin of eye: (0) < 1/4; (1) ≤ 1/2; (2) ≤ 1

24. W of the indentation on the posterior margin of the compound eye/L OT: (0) ≤ L OT; (1) > L OT; (2) ≥ 2x L OT

25. L of the line from the posterior margin of compound eye bisecting the compound eye facets/L OT: (0) absent; (1) <; (2) ≥; (3) ≥ 2x

### Antennae

26. L scape /L pedicel: (0) ≤; (1) ≤ 3x; (2) > 3x

27. L PP/ L pedicel: (0) ≤ 3x; (1) ≤ 4x; (2) > 4x

28. PP base shape: (0) broad not round base; (1) onion-like, round base abruptly narrowed; (2) medially divided, laterally; (3) conical, broad base, gradually narrowed

29. L PP rod/L base: (0) long > 2x; (1) short < 2x; (2) no thin rod

30. Distinct joint between PP and BSM: (0) absent; (1) present

31. L BSM/L pedicel: (0) absent; (1) ≤; (2) ≤ 2x; (3) ≤ 3x; (4) > 3x

32. BSM apical hairs: (0) absent; (1) present

33. L ASM/W BSM: (0) < W BSM, minute spine; (1) > W BSM; (2) > 2x; (3) conical twisted hat

### Thorax

34. Collar Ma: (0) pointed; (1) midstyle; (2) pectinate

35. Scm reflective vestiture: (0) bright; (1) very dull dark; (2) not reflective

36. Ma AN: (0) pointed; (1) midstyle; (2) pectinate

37. L PR bristles/ L PN: (0) > 2x; (1) >; (2) <; (3) absent

38. LT vestiture: (0) bare; (1) some hair; (2) dense hair

39. Ma LT: (0) absent; (1) pointed; (2) midstyle; (3) pectinate

40. MT vestiture: (0) bare; (1) some hair; (2) dense hair

41. L PA bristles/Scu: (0) absent; (1) < 0.5; (2) ≤; (3) >

42. L thorax/Scu: (0) ≤ 2x; (1) ≤ 3x; (2) > 3x

43. Scu vestiture reflective: (0) bright; (1) very dull dark; (2) absent

### Legs

44. C_1_ very long setae: (0) absent; (1) some; (2) dense

45. L forefemur/ L coxa: (0) ≤ 1.5x; (1) ≤ 2x; (2) ≤ 2.5x; (3) > 2.5x

46. Forefemoral spines: (0) absent; (1) short < W femur; (2) long

47. Forefemoral long hairs: (0) absent; (1) some; (2) dense

48. Foretibial spicules: (0) absent; (1) some; (2) dense

49. L foretarsus/ L foretibia: (0) ≥ 1; (1) ≥ 0.75; (2) > 0.5

50. Foretarsal microchaetae: (0) absent; (1) very few < 10; (2) present

51. Foretarsal microchaetae: (0) absent; (1) ends bulbous; (2) ends slightly bent; (3) ends distinctly bent

52. L foreclaw/ L midclaw: (0) < claw; (1) ≤ half; (2) ≤ third

53. Midfemoral spines: (0) absent; (1) short < W femur; (2) some long

54. Midfemoral long hairs: (0) absent; (1) some; (2) dense

55. Midtibial spicules: (0) absent; (1) some; (2) dense

56. L midpulvilli/ L claw: (0) ≥; (1) <; (2) ≤ half; (3) < 0.2

57. Midpulvilli: (0) large, flattened, membranous; (1) small rounded setose; (2) chisel-conical

58. Hindfemoral spines: (0) absent; (1) short < 0.5 W femur; (2) some long

59. Hindfemur long hairs: (0) absent; (1) some; (2) dense

60. Long hindtibial scales: (0) no long scales; (1) some long scales; (2) fluffy - protruding; (3) feathery; (4) very long, dense, feathered fringes

61. Hindtibial spicules: (0) absent; (1) some spicules; (2) apical patch; (3) dense spicules

62. Hindtibial spicules L: (0) absent; (1) same; (2) inner row longer

63. L hindpulvilli/L claw: (0) ≥; (1) <; (2) ≤ half; (3) ≤ 0.2

64. Hindpulvilli: (0) large, flattened, membranous; (1) small rounded setose; (2) chisel -conical

### Wing

65. Patagium: (0) absent hairs only; (1) present scales

66. Basicosta: (0) absent; (1) blunt; (2) sharp

### Wing venation

67. Crossvein forming an extra anterior apical submarginal cell: (0) absent; (1) extra apical submarginal cell

68. R_2+3_ join R_4+5_: (0) basal to r-m > L r-m; (1) at r-m

69. R_2+3_ rises from R_4+5_: (0) acutely; (1) at right angles

70. 2nd r-m crossvein: (0) absent; (1) present

71. Spurvein base R_2+3_: (0) absent; (1) bump or present

72. R_2+3_ apical loop: (0) long apical loop; (1) loop > 180°; (2) at least 90° bend; (3) absent

73. i-r_1_, R_2+3_ to R_4_: (0) absent; (1) present

74. i-r_1_ crossvein: (0) absent; (1) straight; (2) slightly sinuous; (3) distinctly sinuous

75. Spurvein i-r: (0) absent; (1) bump or present

76. L i-r_1_/L r-m: (0) absent; (1) ≤; (2) ≤ 2x; (3) < 3x; (4) ≥ 3x

77. Spur-vein base R_4+5_: (0) absent; (1) bump or present

78. Spur-vein R_4_: (0) absent; (1) bump or present

79. R_5_/R_1_ meet wing: (0) R_5_ distal to R_1_; (1) equal; (2) R_5_ basal to R_1_

80. i-r_2_, R_4_ to R_5_: (0) absent; (1) present

81. M_1_: (0) straight; (1) slightly sinuous; (2) sinuous

82. L m-m/r-m: (0) ≤ 2x; (1) ≤ 3x; (2) > 3x

83. m-m: (0) straight; (1) slightly sinuous; (2) sinuous

84. m-m spurvein: (0) absent; (1) into m_2_; (2) into discal; (3) crossvein form basal cell

85. m-m to hind wing margin: (0) oblique; (1) parallel; (2) horizontal

86. M_2_: (0) straight; (1) slightly sinuous; (2) sinuous

87. r_5_ open: (0) open; (1) narrow < r-m; (2) just closed; (3) closed and stalked - acute; (4) closed and stalked -obtuse

88. m_1_ open: (0) open; (1) closed and stalked

89. m_2_ open: (0) open; (1) closed and stalked - acute

90. L mcu/r-m: (0) > 3x; (1) < 3x; (2) ≤ 2x; (3) ≤

91. m-cu: (0) straight; (1) slightly sinuous; (2) 90° basally; (3) sinuous

92. Spur-vein m-cu: (0) absent; (1) spur into discal cell

93. Spur-vein into M_2_: (0) absent; (1) spur-vein into m_2_; (2) cross-vein to CuA_1_

94. W anal/ W posterior cubital: (0) ≤; (1) ≤ 1.5x; (2) ≤ 2x; (3) > 2x

95. cup open: (0) open; (1) narrow < r-m; (2) closed at wing margin (Fig. 9B); (3) closed and stalked - acute

96. Anal lobe margin: (0) hairs; (1) some scales; (2) scales dense

97. Anal cell: (0) broad rounded; (1) rounded; (2) thin linear; (3) very reduced

98. Alula reduced: (0) not reduced; (1) reduced, L < 4x W

99. Alula margin: (0) hairs; (1) some scales; (2) scales dense

100. Squamal margin: (0) hairs; (1) some scales; (2) scales dense

101. Squama reduced: (0) not reduced; (1) reduced, L < 4x W

102. wing L: (0) ≤ 10; (1) 10-15; (2) 16-20; (3) 21-25; (4) > 25

103. wing L/W: (0) ≤ 3x; (1) long > 3x

104. L wing/ L abdomen: (0) ≥ 3x; (1) ≥ 2 x; (2) > 1.5 x; (3) >

### Abdomen

105. Abdomen apically: (0) rounded; (1) narrowed; (2) truncate, parallel sided

106. Abdomen L/ W T_2_: (0) > 2.5x; (1) < 2.5x; (2) < 2x; (3) ≤ 1.5x; (4) ≤

107. Abdominal vestiture reflective: (0) bright; (1) very dull dark; (2) not reflective

108. Abdominal bristles/hair: (0) dorsally and laterally; (1) lateral and apically T_7_; (2) apically; (3) absent

109. Long lateral hairs > T_1_: (0) dense tufts; (1) some; (2) absent

110. Ma T_1_: (0) pointed; (1) midstyle; (2) pectinate

111. Scales: (0) absent; (1) adpressed scales; (2) upstanding scales; (3) long upstanding scale tufts

### Male genitalia

112. Male genitalia twisted: (0) no twisting, gonocoxae ventral; (1) 90°; (2) 180°, gonocoxae dorsal

113. E setae grouping: (0) not grouped; (1) medioapically; (2) lateroapically; (3) laterally

114. E setae group: (0) not grouped; (1) loose; (2) strong; (3) dense tufts

115. Epandrial spines: (0) absent; (1) short and broad; (2) long

116. E apically: (0) deeply indented; (1) concave-indented; (2) truncate; (3) convex rounded; (4) convex pointed

117. E apical flange: (0) absent; (1) < quarter base; (2) < third base; (3) < half base; (4) > half rest base

118. E medial flange: (0) absent; (1) slight; (2) distinct < quarter base

119. L E basal flange: (0) absent; (1) < quarter base; (2) < third base (Fig. 10B); (3) < half base; (4) ≥ half rest base; (5) > base

120. Mid W/L basal flange: (0) absent; (1) < quarter length base; (2) < half length; (3) > half length (4) > length

121. E posterolateral flange: (0) absent; (1) < quarter length base

122. E basal flange recurved: (0) absent (Fig. 10B); (1) < quarter base; (2) < third base; (3) < half base; (4) > half rest base; (5) > base

123. E basally extended: (0) absent; (1) present

124. SES: (0) absent; (1) linear; (2) triangular; (3) quadrate; (4) single (Fig. 10A)

125. L SES/G W: (0) absent; (1) < eighth; (2) < quarter; (3) > quarter

126. G setae: (0) some; (1) dense; (2) tufts

127. G setae group: (0) absent; (1) not grouped; (2) apically; (3) medially; (4) laterally; (5) basally

128. Thick G setae number: (0) absent; (1) no thick setae; (2) some; (3) many; (4) 6-8 long

129. Thick G setae position: (0) absent; (1) no thick setae; (2) apically; (3) medially; (4) laterally; (5) basally

130. G subapical indentation: (0) absent; (1) slight < third; (2) narrowed apically > third

131. W G medial indentation: (0) absent; (1) < third; (2) > third; (3) > half

132. G medial weakness: (0) absent; (1) desclerotised line; (2) lines of weakness; (3) division medially

133. G ventral division: (0) line fusion basally; (1) line fusion entire; (2) fused medially; (3) fused basally; (4) fused

134. G medioventrally: (0) deeply indented; (1) indented medially; (2) flat; (3) convex shell

135. G ventral ridge: (0) absent; (1) slight; (2) distinct

136. G basal projection of the ventral ridge: (0) absent; (1) slight; (2) distinct; (3) recurved hook

137. G basomedial margin: (0) deeply indented; (1) indented, concave; (2) smooth, linear; (3) convex

138. H: (0) absent; (1) present

139. H laterally: (0) absent; (1) indented between G, smooth; (2) projecting; (3) with spur; (4) with finger (Fig. 10F)

140. RM: (0) small; (1) > L GS; (2) large recurved

141. L G A: (0) absent; (1) < GS; (2) > GS

142. G plates dorsoapically: (0) medially parallel; (1) angled basomedial plates diverge dorsally

143. G dorsoapical plates extension apically: (0) not extended apically; (1) small apical extension; (2) long apical extension beyond the base of the gonostyli

144. GS: (0) large base, long pointed flange; (1) laterally bifid; (2) simple curved hook; (3) medial hook; (4) lateral hook

145. GS basal projection: (0) absent; (1) small; (2) < GS

146. L AE: (0) < GS; (1) = GS; (2) > GS

147. AE EP separate: (0) absent; (1) present

148. EP: (0) absent; (1) present

149. EP deep ventral notch: (0) no EP; (1) absent; (2) medial

150. EP expanded apically: (0) no EP; (1) not expanded (Fig. 10H); (2) ≤ 2x neck; (3) < 3x neck; (4) > 3 × neck

151. EP apical plate: (0) no EP; (1) absent; (2) apical plate

152. EP medioventral projection: (0) no EP; (1) absent; (2) above AE

153. EP lateroapical lobes: (0) no E; (1) absent; (2) rounded dorsally; (3) pointed dorsally

154. EP lateral projection laterally: (0) no EP; (1) absent (2) lateral

155. EP medial projection laterally: (0) no EP; (1) absent; (2) medial

156. EP pair ventral projections: (0) no EP; (1) absent; (2) medial below AE

157. L EP: (0) no EP; (1) < GS base; (2) > GS base; (3) > G margin

158. EP recurved apicomedial projection: (0) no EP; (1) absent; (2) present dorsally

159. EP recurved apically: (0) no E; (1) absent; (2) apex recurved lateral view

160. EP apical setae: (0) no EP; (1) absent; (2) present

161. BP expanded: (0) not expanded; (1) round; (2) swollen spherical; (3) bilobed

162. LAEA: (0) spoon convex up; (1) spoon concave; (2) linear; (3) absent

163. L lateral AE A: (0) < L GS; (1) < G margin; (2) = G margin; (3) absent

164. AAES: (0) spoon convex; (1) spoon concave; (2) narrow wedge; (3) linear

165. AAES: (0) < B; (1) = L GS; (2) < G margin; (3) to G margin

166. EJA: (0) racquet - round; (1) linear

167. L EJA: (0) within G; (1) = G; (2) > G < L GS; (3) > G > L GS

### Female genitalia

168. AC spines: (0) 3 prs; (1) 4 prs; (2) 5 prs; (3) > 5 prs; (4) > 10 prs

169. AC spines apically: (0) thin tapering; (1) broader spoon shaped

170. T_9+10_ sclerites: (0) 1 sclerite; (1) 3 sclerites

171. T_9_ dorsal medioapical unsclerotised lacuna: (0) absent; (1) present

172. T_8_ dorsal medioapical unsclerotised lacuna: (0) absent; (1) present

173. T_8_ hair: (0) apical half; (1) apical half bare medially; (2) apical edge

174. T_8_ laterally: (0) not indented; (1) indented arms

175. T_8_ A divided medially: (0) not divided; (1) slightly; (2) distinctly ≥ half width

176. T_8_ A lateral projections: (0) absent; (1) slight; (2) not linear; (3) linear

177. T_8_ A L/medial W: (0) margin thickened, sclerotised; (1) ≤ quarter; (2) ≤ half; (3) <; (4) ≥; (5) > 2x; (6) ≥ 3x

178. T_8_ A internal structure: (0) absent; (1) linear; (2) quadrate plate

179. S_8_ sclerites: (0) 1 linear 2 round; (1) 1 linear 2 round 1 medial; (2) U-shaped 2 triangular; (3) 2 round; (4) sheet

180. Furca: (0) U-shaped; (1) 3 separate sclerites; (2) 2 rods

181. ST tube: (0) short < third L pump; (1) present; (2) long > × 8 L pump

182. Basal endplate: (0) absent; (1) small; (2) present (Fig. 9H); (3) large

183. Basal endplate: (0) absent; (1) simple-thin processes; (2) thick processes; (3) funnel

184. Sperm pump: (0) short pump; (1) very long pump

185. Long pump papillae: (0) no long papillae; (1) unpigmented; (2) pigmented

186. Pump processes basally: (0) no processes; (1) short processes; (2) long papillae

187. Pump processes medially: (0) no processes; (1) short processes; (2) long papillae

188. Pump processes apically: (0) no processes; (1) short processes

189. Apical endplate: (0) large; (1) present; (2) small; (3) absent

190. Apical endplate: (0) absent; (1) simple- thin processes; (2) thick processes; (3) funnel; (4) double

191. SR: (0) L ≤ W; (1) L > W; (2) L > 2 W; (3) L > 4 W; (4) L > 6 W; (5) L > 8 W; (6) L > 30 W

192. ST basal sclerotised plate: (0) absent; (1) basal sclerotised plate

193. Tube ST to pump: (0) absent; (1) present; (2) long

194. SR shape: (0) round square; (1) oval; (2) pear, expanded apically; (3) long; (4) expanded basally

195. SR apically: (0) rounded blunt; (1) nipple; (2) narrowed; (3) knob

196. ST round basal bulb: (0) no round BB; (1) round BB

197. SR pigmented: (0) unpigmented; (1) pigmented; (2) basally unpigmented

198. ST long medial tube: (0) no medial tube; (1) tube medially

199. Long membranous base: (0) no long base; (1) long MB

200. ST long MB basally swollen: (0) no long base; (1) symmetrically; (2) asymmetrically

201. ST clear rings: (0) no rings; (1) clear ring; (2) long striated collar

202. SR to pump: (0) symmetrical; (1) asymmetrical

203. SR medially bent: (0) absent; (1) bent reservoir

204. SR apically bent: (0) absent; (1) tip only

205. Tubules: (0) absent; (1) present

206. SR walls: (0) thin unsclerotised; (1) thick sclerotised

207. SR walls: (0) no dimples; (1) with dimples thin unsclerotised
